# Circular RNA Circ-03955 Promotes Epithelial-Mesenchymal Transition in Osteosarcoma by Regulating miR-3662/Metadherin Pathway

**DOI:** 10.3389/fonc.2020.545460

**Published:** 2020-11-18

**Authors:** Zhengguang Wang, Mingsi Deng, Liangjian Chen, Weiguo Wang, Gengyan Liu, Dongbiao Liu, Zhipeng Han, Yong Zhou

**Affiliations:** ^1^ Department of Orthopaedics, The Third Xiangya Hospital, Central South University, Changsha, China; ^2^ Department of Stomatology, The Third Xiangya Hospital, Central South University, Changsha, China

**Keywords:** circ-03955, miR-3662, osteosarcoma, metadherin, epithelial-mesenchymal transition

## Abstract

Osteosarcoma is the most common primary malignant tumor, especially in children and adolescents. Circular RNAs (circRNAs) are found to play roles in the progression of osteosarcoma. However, the exact functions of circRNAs in osteosarcoma development still need to be clarified. We obtained differentially expressed circRNAs and miRNAs from a GSE99671 data set (GEO database). The gene co-expression network of ceRNAs and osteosarcoma-related genes was analyzed using the STRING database. qRT-PCR was used to detect the expression of circ-03955 and miR-3662. Transwell assays and flow cytometry were performed to detect phenotypic changes in cell function. A xenograft tumor model was established using BALB/c nude mice. Dual luciferase activity and RNA immunoprecipitation assays were performed to assess the relationship between circ-03955, miR-3662, and metadherin (MTDH). Immunohistochemistry, immunofluorescence, and Western blotting were used to assess protein expression levels. Circ-03955 was significantly upregulated, and miR-3662 was downregulated in osteosarcoma. Circ-03955 silencing inhibited the growth and metastasis of osteosarcoma. Mechanism analysis revealed that circ-03955 could bind to miR-3662, and the latter could target MTDH, leading to its suppressed expression and facilitating epithelial-mesenchymal transition (EMT). All these findings demonstrate that the presence of circ-03955 promotes EMT in osteosarcoma by acting as miR-3662 sponge-mediated MTDH expression.

## Introduction

Osteosarcoma is the most common malignant bone neoplasm, especially in children and adolescents. It has a high rate of mortality and disability (1, 2) as well as high levels of metastasis and invasion. At present, the treatment of osteosarcoma usually involves a combination of chemotherapy and surgical resection. The five-year survival rate of non-metastatic osteosarcoma patients is 60%−70% (3), but the five-year survival rate of patients with distant metastasis is only 20% (4). Therefore, it is important to explore the molecular mechanisms of osteosarcoma occurrence and metastasis, find molecular biomarkers for early diagnosis, and identify an effective molecular basis for the development of novel therapeutic targets in order to inhibit the occurrence of osteosarcoma metastasis.

Circular RNA (circRNA) is a relatively newly identified form of RNA. The 3’ and 5’ ends of circRNA are linked together, forming a closed ring structure, and circRNA is abundant in the eukaryotic transcriptome. In 2012, Salzman (5) found that many highly expressed circRNAs are associated with their corresponding linear RNA, which can form multiple homotypes of circular RNA by “non-classical splicing,” and speculated that the expression of circRNA might be a general pattern of gene expression program in human cells. There is increasing evidence that abnormal circRNA plays an important role in tumorigenesis (6, 7), tumor development (8, 9), and metastasis (10, 11). Chen confirms that circ-100782 is highly expressed in pancreatic ductal adenocarcinoma and regulates BxPC3 cell proliferation through the IL6-STAT3 pathway (12). Studies show that circRNA can regulate the proliferation and apoptosis of osteosarcoma and are good targets for clinical diagnosis (13–15). However, the effect of circRNA on osteosarcoma has not been studied in detail.

Current studies show that miRNAs can be involved in the regulation of gene expression and can complement the transcripts of target genes, thus playing an important role in the occurrence and development of tumors (16, 17). The biological function of circRNA is related to that of miRNAs. A regulatory network is formed by competitive inhibition. Li shows that circ-0001785 regulates the pathogenesis of osteosarcoma by sponging miR-1200, which upregulates HOXB2 expression (18). Song indicates that hsa_circ-0001564 acts as an miR-29c-3p sponge to mediate the tumorigenicity of osteosarcoma (19). More details about the function of ceRNA in osteosarcoma still need to be elucidated.

In this study, we first screened circRNA (circ-03955) from the GEO database (GSE99671 data set) and find that circ-03955 was highly expressed in osteosarcoma. We also find that circ-03955 knockdown could inhibit the migration, invasion, and epithelial-mesenchymal transition (EMT) of osteosarcoma. In addition, circ-03955 acts as a ceRNA for miR-3662 to regulate the progression of metadherin (MTDH)-mediated osteosarcoma.

## Materials and Methods

### Patient Tissue Specimens

Forty samples of osteosarcoma tissue and 40 cases of adjacent non-osteosarcoma tissues were obtained from the Department of the third Xiangya Hospital of Central South University. All tissue specimens were removed during scheduled surgery. After rinsing the tissue specimens with saline, the specimens were cut into small pieces and frozen in liquid nitrogen. The specimens were then stored at −80°C for subsequent RNA extraction experiments. The use of normal bone tissue specimens and osteosarcoma bone tissue specimens in all patients was evaluated and approved by the Ethical Committee of the third Xiangya Hospital of Central South University. All participants provided written informed consent after receiving information about the design of the study.

### Cell Culture and Transfection

A human osteoblastic cell line, OB3, and the human osteosarcoma cell lines Saos-2, MG-63, and U2OS were purchased from the American Type Culture Collection (ATCC, Rockville, USA). Saos-2 and U2OS cells were cultured in McCoy’s 5a medium modified (ATCC, USA), but 15% fetal bovine serum was added to Saos-2 growth medium, and 10% fetal bovine serum was added to MG-63 growth medium. MG-63 cells were cultured in Eagle’s minimum essential medium (ATCC, USA) containing 10% heat-inactivated fetal bovine serum. The circ-03955 interference vectors sh-circ-03955-1 and sh-circ-03955-2 and the circ-03955 overexpression vector pCDNA3.1-circ-03955 (OE-circr-03955) were obtained from GenePharma (Shanghai, China). A non-targeting shRNA (shNC) or the empty pcDNA3.1vector (OE-NC) were used as negative controls. The miR-3662 mimics and miR-3662 inhibitor were purchased from GeneCopoeia (Guangzhou, China). The MTDH mRNA interference vector sh-MTDH was obtained from GenePharma (Shanghai, China). All transfections were performed using Lipofectamine 6000 reagent (Life Technologies, USA) according to the manufacturer’s protocols.

### Bioinformatic Analysis

The GSE99671 data set, which included 18 pairs of osteosarcoma tissue specimens and paired normal bone tissue specimens, from the GEO database (https://www.ncbi.nlm.nih.gov/geo) was analyzed for differential gene expression. Gene expression was calculated using | log2FC | > 1 and FDR < 0.05 as the criteria for significant difference using the R statistics package limma. The miRanda package was used to predict the miRNAs that can bind MTDH and circRNA, and then the possible ceRNA network of differentially upregulated circRNA, differentially downregulated miRNAs, and MTDH was constructed. The first 20 genes reported to be associated with osteosarcoma were selected from the DisGeNET database for co-expression analysis with the ceRNA network maps. Cytoscape 3.6.1 was used to build the network graph.

### Quantitative Reverse Transcription-Polymerase Chain Reaction Assay

The linear validation of circRNA was performed in the osteosarcoma cell line MG-63. MG-63 cells were extracted using Trizol reagent (Invitrogen, California, USA), and circRNA was linearized by RNase R. Then, the expression of circular RNA was detected using qRT-PCR assay. The specific steps were carried out as previously described (20). SYBR Green I fluorescent dye was used in the qRT-PCR assays. The relative expression of these genes was calculated with triplicate experiments using the 2^–ΔΔCT^ equation. The following primers were used for qRT-PCR detection. Primer mRNA (Forward: 5’-TGCTCCACTGACTGTTGT-3’; Reverse: 5’-TTGGCATTTCTCCTCTAA-3’), primer circ-03955 (Forward: 5’- ATTAGAGGAGAAATGCCAAGG-3’; Reverse: 5’-AGCCCATCTGCAACAACAG-3’), primer miR-3662 (Forward: 5’-CGCTCACAGTTACACTTCTT-3’; Reverse: 5’-GTGCTTCATCAGTCACTACTCATC-3’), U6 (Forward: 5’-CTCGCTTCGGCAGCACA-3’; Reverse: 5’-AACGCTTCAGGAATTTGCGT-3’), and the internal reference primer GAPDH (Forward: 5’-CCTTCCGTGTTCCTAC-3’; Reverse: 5’-GACAACCTGGTCCTCA-3’). All primers were designed and synthesized by Sangon Biotech (Shanghai, China).

### Cell Proliferation

Cells were digested with 0.25% trypsin and suspended in basic culture medium containing 10% fetal bovine serum to form a single-cell suspension. The single-cell suspension was inoculated into 96-well plates at a concentration of 1 × 10^5^/well. The culture plate was placed in an incubator at 37°C containing 5% CO_2_ and saturated humidity. The suspension of cells was centrifuged at 2000 rpm for 15 minutes. The supernatant was dissolved and crystallized by adding 150 μL DMSO. The cells were transfected for 24 hours by enzyme-linked immunosorbent assay (Bio-Rad, USA). The OD value at 570 nm was detected after 24, 48, and 72 hours of transfection.

### Cell Apoptosis

The cells were digested with 0.25% trypsin, and then the digested cells were combined into a single-cell suspension (1 × 10^6^/mL) in basic culture medium containing 10% fetal bovine serum. After centrifugation with 1000 rpm for five minutes, the culture medium was discarded, the cells washed with precooled PBS (Solarbio, Beijing, China) and centrifuged at 1000 rpm for 5 minutes. The cells were resuspended in 100 μL of binding buffer (1 × 10^5^/mL), lightly blended with 5 μL Annexin V-FITC at room temperature for 10 minutes and then incubated with 5 μL PI at room temperature for 5 minutes. PBS was then added to 500 μL, and the apoptotic rate was detected immediately after blending.

### Cell Migration and Invasion

Serum was removed to starve the cells for 24 hours, and 0.25% trypsinase was added to digest the cells. After termination of digestion, the cells were washed twice with precooled PBS and then suspended in serum-free medium containing BAS, and the cell concentration was adjusted to 5 × 10^5^/mL. A 100-μL cell suspension was added into Transwell chambers, and 600 μL of medium containing 10% FBS was added into a 24-well plate subchamber. After 24 hours of culture in a constant temperature incubator with 5% CO_2_ and saturated humidity of 37°C, the Transwell chamber was removed, and the culture medium in the well was washed twice with PBS and then fixed with methanol for 30 minutes. The upper chamber liquid was removed, and the cells dyed with 0.1% crystal violet dye solution for 20 minutes. The untransferable cells were wiped on the bottom membrane surface of the upper chamber with a wet cotton rod and washed three times with PBS. Finally, the chamber membrane was observed at 100X magnification. The difference between the invasion and migration assays was that 1 mg/mL of Matrigel (BD, NJ, USA) was added to the bottom of the upper chamber and incubated in a 37°C incubator for 4 hours after dilution by 100 μL. The gelatinized material was then removed and reserved.

### Xenograft Assay

Ten 4- to 5-week old SPF male BALB/c nude mice were purchased from Auragene (Changsha, China). Nude mice were randomly divided into an sh-NC group and an sh-circ-03955 group with five mice in each group. Osteosarcoma MG-63 cells transfected with sh-circ-03955 and sh-NC plasmids were administered by subaxillary injection to form tumors at a cell concentration of 2 × 10^5^ cells. After the osteosarcoma MG-63 cells were injected into the mice, the tumor volume was measured using calipers every five days. Six weeks later, the mice were euthanized, and the tumors were weighed. The study was approved by the medical ethics committee of the third Xiangya Hospital, central South University.

### Immunofluorescence

Logarithmic-stage growth cells of different treatment groups were washed twice with PBS and then used to prepare cell smears. The cells were fixed at room temperature with 4% paraformaldehyde for 30 minutes, washed twice with PBS, and then treated at room temperature with 0.1% Triton-X 100 for 3 minutes to make them permeable. After washing the permeate, 5% skimmed milk powder was added and incubated at room temperature for 2 hours. Anti-E-cadherin antibody (1:300, ab76055, Abcam) and anti-N-cadherin antibody (1:200, ab98952, Abcam) was added and the reaction incubated overnight at 4°C. After the first antibody was washed out, fluorescently labeled goat antimouse IgG antibody (1:200, ab150115, Abcam) was added and incubated at room temperature for 1 hour, then washed with PBST three times. After adding DAPI staining and washing out the staining solution the cells were observed under the fluorescence microscope (Olympus, Japan).

### Western Blotting

Tissues or cells were removed, and then total protein was extracted using RIPA buffer. BCA protein concentration kits (Sigma-Aldrich, USA) were used to determine the protein concentration. SDS sample buffer was added, and the reaction mixture was placed in a 95°C water bath for 5 minutes, then centrifuged after cooling. Electrophoresis using 10% SDS-PAGE gels was performed with 20 μg protein per well. After the completion of electrophoresis, the protein obtained was transferred to PVDF transfer membranes using a wet rotation method. The protein was sealed at room temperature for one hour with 5% BSA (Solarbio, Beijing, China). Then antibodies to MTDH (1:1000, 9596, CST), E-cadherin (1:1000, 3195T, Cell Signaling Technology), N-cadherin (1:1000, 13116T, Cell Signaling Technology), Vimentin (1:1000, 5741T,Cell Signaling Technology), GAPDH (1:500, ab8245, Abcam), and β-actin (1:500, ab8226, Abcam) were added to the membrane. After incubation overnight at 4°C, the membranes were washed three times with TBST buffer. Membranes were incubated at room temperature for 30 minutes with goat antimouse IgG antibody labeled with horseradish peroxidase and then washed with TBST buffer three times. Color was developed using immunoblotting chemiluminescence reagent ECL (Auragene, Changsha, China) and then detected using a gel imaging system.

### Immunohistochemistry

Immunohistochemistry (IHC) was performed to evaluate changes in the expression levels of E-cadherin and N-cadherin. The xenograft tissue sections of the sh-NC and sh-circ-03955 groups were taken out and completely dewaxed and hydrated. The antigens were repaired by microwave. Then, the sections were washed with PBS three times. The sections were immersed in 3% H_2_O_2_-methanol solution for 15 minutes at room temperature to block endogenous peroxidase and then washed with PBS three times. E-cadherin (1:500, GB12082, Servicebio) and N-cadherin (1:800, GB11135, Servicebio) were added and incubated in a 37°C water bath for one hour, followed by post-blocking incubation at room temperature for 30 minutes and then washing with PBS three times. The second antibody labeled with horseradish peroxidase was incubated at room temperature for 30 minutes and washed with PBS five times. After adding DAB for two minutes, the dyeing was terminated, re-dyed with hematoxylin for three minutes, and then sealed with transparent xylene and dripped with neutral gum. Under the light microscope, positive brown staining was observed at a magnification of 200×.

### Dual Luciferase Activity Assay

MG-63 cells were transfected with Wt-circ-03955 plasmid and NC mimics or miR-3662 mimics, according to the instructions accompanying the Lipofectamine 6000 transfection reagent. Mut-circ-03955 plasmid and mimics of NC or miR-3662 were co-transfected, and lysate was added 48 hours later. The supernatant was centrifuged for 10 minutes at 4°C at 12,000 rpm, and the luciferase activity was detected using a Dual-Luciferase^®^ Reporter Assay System (Promega, Wisconsin, USA). MTDH binding to miR-3662 was detected in a similar way.

### RNA Immunoprecipitation (RIP) Assay

MG-63 cells (2 × 10^7^) were collected and added to the same volume of RIP lysate to lyse cells. The supernatant was centrifuged at 12,000 rpm for 10 minutes. In accordance with the instructions of the RIP™ RNA binding protein immunoprecipitation reaction kit, 900 μL of RIP immunoprecipitation buffer containing RNase inhibitor, protease inhibitor, and DNase and 100 μL of cell lysate were added into EP tubes containing magnetic beads. IgG antibody or Ago2 antibody were added and incubated overnight at 4°C and centrifuged at 12,000 rpm. After centrifugation for 10 minutes, the supernatant was discarded and washed six times with 500 μL RIP wash buffer. The RNA was purified immediately, and then dissolved and purified in 15 μL of DEPC water, then stored at −80°C. The positive control (input) group had no antibody, and the experimental group had Ago2 antibody. The expression of circ-03955 and microRNA-3662 in the input group was detected using qRT-PCR assay.

### Statistical Analysis

SPSS19.0 statistical software and GraphPad Prism 6.0 software were used to analyze all the data. Student’s *t-*test was used to analyze the differences between the two groups. One-way ANOVA was used to analyze the differences between more than two groups. All of the experimental data are presented as mean ± standard deviation (SD). Chi-square tests were used to analyze the correlations between the expression of miR-3662 and circ-03955 in osteosarcoma cells. The *P* value was evaluated to determine statistical significance with a *P* value of less than 0.05 taken to indicate that the difference was statistically significant.

## Results

### Screening of miR-3662 and circ-03955 by Bioinformatic Analysis

**Figure 1:**
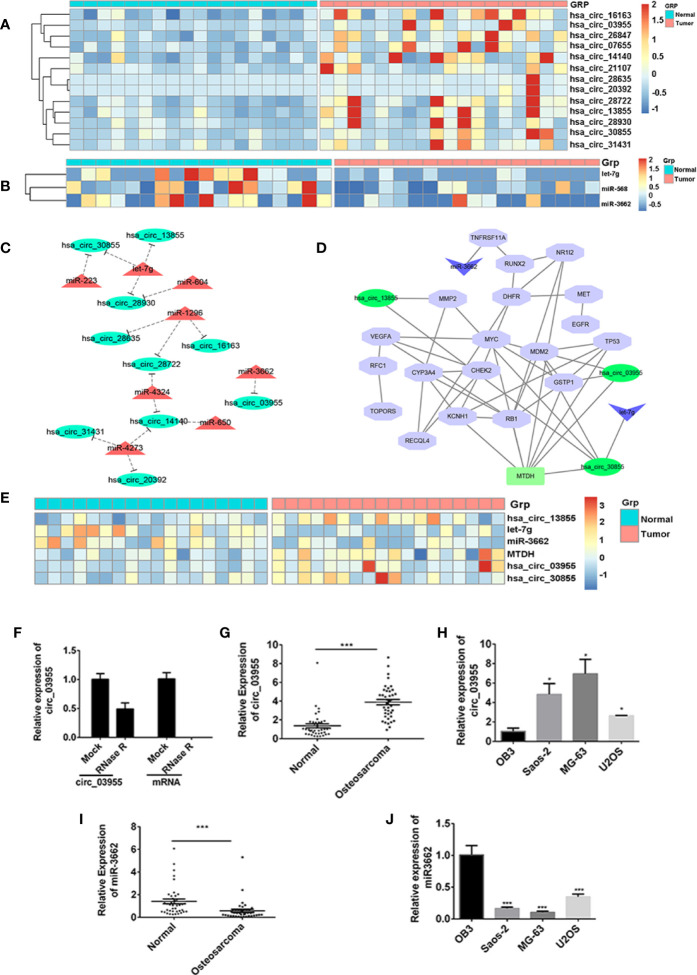
circ-03955 was markedly increased in osteosarcoma cells and tissues. **(A)** Thermal map of circRNAs that are differentially expressed in the GSE99671 data set. **(B)** Thermal map of the miRNAs that regulate MTDH expression and are differentially downregulated in the GSE99671 data set. **(C)** Network map constructed using the GSE99671 data set showing significantly upregulated circRNA-bound miRNAs and downregulated miRNAs. **(D)** The first 20 genes associated with osteosarcoma were screened by the DisGeNET database for co-expression analysis with ceRNA network maps. **(E)** Thermograph of gene expression in the ceRNA network of Fig 1D, which was analyzed using GSE99671. **(F)** The expression of circ-03955 and mRNA detected by qRT-PCR in MG-63 cells treated with RNase R. **(G)** The expression of circ-03955 detected by qRT-PCR in 40 samples of osteosarcoma tissue specimens and 40 samples of adjacent non-osteosarcoma tissue specimens. **(H)** Expression of circ-03955 detected by qRT-PCR in normal osteoblasts OB3 in three osteosarcoma cell lines: Saos-2, MG-63, and U2OS. **(I)** The expression of miR-3662 detected by qRT-PCR in 40 samples of osteosarcoma tissue specimens and 40 samples of adjacent non-osteosarcoma tissue specimens. **(J)** Expression of miR-3662 detected by qRT-PCR in normal osteoblasts OB3 and three osteosarcoma cell lines: Saos-2, MG-63, and U2OS. Data are presented as the mean ± standard deviation of three independent experiments, *p < 0.05, ***p < 0.001 vs. normal group or OB3 group.

To screen differentially expressed miRNA and circRNA in osteosarcoma, we performed bioinformatic analysis on data from the GSE99671 data set, comprising 18 pairs of osteosarcoma tissue specimens and paired normal bone tissue specimens. We identified nine downregulated miRNAs (**Table 1**) and 13 upregulated circRNAs (**Figure 1A**) compared with normal bone tissue. Then, we used the miRanda software to predict differentially downregulated miRNAs in osteosarcoma bone samples that can interact with MTDH, including miRNA-563 and miR-3662 (**Figure 1B**). The miRanda software was also used to predict eight miRNAs that could bind to significantly upregulated circRNA and were differentially downregulated in osteosarcoma bone samples. Cytoscape 3.6.1 was used to construct a network map (**Figure 1C**). The DisGeNET database was used to select the first 20 reported osteosarcoma-related genes: CHEK2, TP53, RB1, VEGFA, EGFR, RUNX2, MDM2, MMP2, MET, DHFR, TNFRSF11A, JUN, RFC1, RECQL4, GSTP1, NR1I2, TOPORS, CYP3A4, MYC, and KCNH1. The ceRNA network map was analyzed using co-expression analysis, and the results show that MTDH and circ-03955 play an important role in the graph (**Figure 1D**). The expression of circ-03955, MTDH, and miR-3662 was found to be inversely correlated in osteosarcoma tissues (**Figure 1E**).

**Table 1:** Differential expression of miRNAs in 18 osteosarcoma bone samples and 18 paired normal bone samples.

Gene ID	log2FC	pvalue
miR1296	-1.616	0.00053
LET7G	-1.191	0.01551
miR4273	-0.832	0.04439
miR568	-0.880	0.02349
miR3662	-0.925	0.04806
miR604	-0.956	0.04127
miR4324	-1.439	0.00352
miR650	-1.236	0.00543
miR223	-1.087	0.02862

### High Expression of circ-03955 in Osteosarcoma Cells and Tissues

To verify the differential expression of circ-03955 and miR-3662 in osteosarcoma tissues and cells, we first linearized them using RNase R in MG-63 cells, and then detected the expression of circ-03955. The results show that circ-03955 is resistant to RNase R digestion, and linear circ-03955 is easy to degrade. They further confirm that circ-03955 is circular (**Figure 1F**). Previous bioinformatics analysis showed that circ-03955 was highly expressed in osteosarcoma tissue. In this study, we measured the expression of circ-03955 in 40 pairs of osteosarcoma tissues and normal tissues as well as in osteosarcoma cells and normal osteoblasts. The results show that the expression of circ-03955 is significantly increased in osteosarcoma bone tissue compared with adjacent non-osteosarcoma tissue (**Figure 1G**). The same results were also found in cells in which the expression of circ-03955 in osteosarcoma cells from the Saos-2, MG-63, and U2OS cell lines was significantly higher than that in normal osteoblast cells OB3 (**Figure 1H**). MG-63 cells had the highest levels. The expression of miRNA-3662 in osteosarcoma tissue was significantly lower than that in adjacent non-osteosarcoma tissue (**Figure 1I**), and the same result was also found in cells (**Figure 1J**).

### circ-03955 Knockdown Suppressed Osteosarcoma Cells Progression *In Vivo* and *In Vitro*

**Figure 2:**
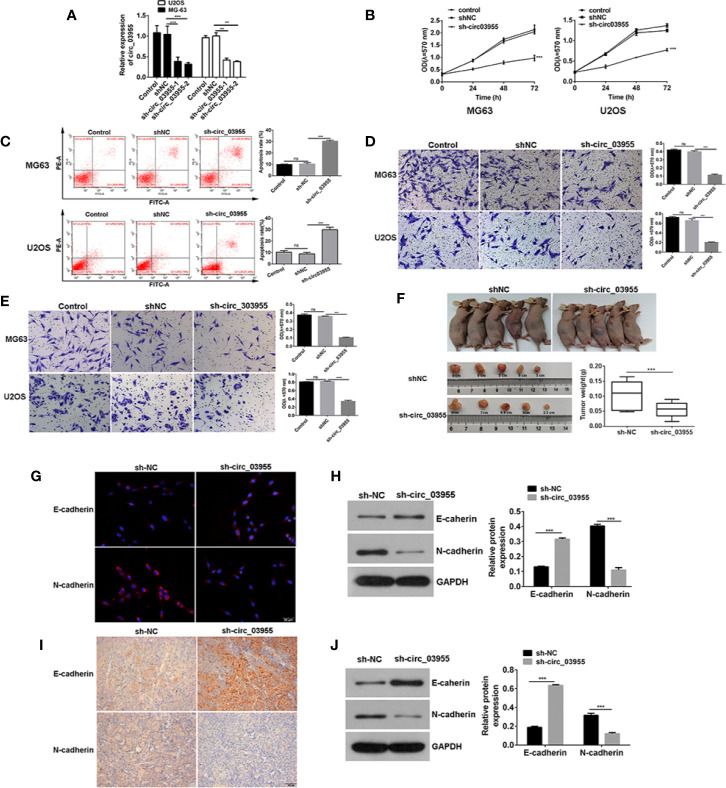
Circ-03955 silencing suppressed osteosarcoma cells progression *in vivo* and *in vitro*. **(A)** qRT-PCR was used to verify whether sh-circ-03955 was successfully transfected into osteosarcoma cells in MG-63 and U2OS. **(B)** MTT assay was used to detect the effects of sh-circ-03955 on the proliferation of osteosarcoma cells in MG-63 and U2OS at 24, 48, and 72 hours after transfection. **(C)** Flow cytometry was used to detect the effect of sh-circ-03955 on the apoptosis of osteosarcoma cells in MG-63 and U2OS 48 hours after transfection. **(D)** Transwell assay was used to detect the effects of sh-circ-03955 on the migration of osteosarcoma cells in MG-63 and U2OS at 48 hours. **(E)** Transwell assay was used to detect the effect of sh-circ-03955 on invasion of osteosarcoma cells in MG-63 and U2OS at 48 hours. **(F)** Size and weight of transplanted tumors. **(G)** The expression of E-cadherin and N-cadherin in osteosarcoma MG-63 cells transfected with sh-NC and sh-circ-03955 for 48 hours was detected by immunofluorescence. **(H)** Western blotting was used to detect the expression of E-cadherin and N-cadherin in osteosarcoma MG-63 cells transfected with sh-NC and sh-circ-03955 for 48 hours. **(I)** After transfection of osteosarcoma MG-63 cells with sh-NC and sh-circ-03955, the tumorigenesis experiment was carried out in nude mice, and the transplanted tumor tissues were removed and immunohistochemical assays were performed. (Scale bar = 500 μm, 200×) **(J)** After transfection of osteosarcoma MG-63 cells with sh-NC and sh-circ-03955, the tumorigenesis experiment was carried out in nude mice, and the transplanted tumor tissues were removed for Western blot detection. Data are presented as the mean ± standard deviation of three independent experiments, NS, not significant, **p < 0.01, ***p < 0.001 vs. shNC group.

To verify the effect of circ-03955 on osteosarcoma, we constructed a circ-03955 interference expression vector and transfected it into MG-63 and U2OS cells (**Figure 2A**). Subsequently, the proliferation of MG-63 and U2OS cells in the sh-circ-03955 group decreased significantly compared with the sh-NC group (*P <* 0.001) (**Figure 2B**). Compared with the sh-NC group, the apoptotic rate of MG-63 and U2OS cells in the sh-circ-03955 group was significantly higher (*P <* 0.001) (**Figure 2C**). Compared with the sh-NC group, the migration of MG-63 and U2OS cells in the sh-circ-03955 group was significantly decreased (***P < 0.001) (**Figure 2D**). Compared with the sh-NC group, the invasion of MG-63 and U2OS cells in the sh-circ-03955 group was significantly decreased (*P <* 0.001, *P <* 0.001) (**Figure 2E**).

Assays in xenograft mice showed that circ-03955 knockdown not only suppressed tumor volume, but also inhibited tumor weight, suggesting a suppressor role for circ-03955 knockdown on osteosarcoma tumor growth (**Figure 2F**). Overall, these results indicate that circ-03955 silencing could suppress the osteosarcoma cells progression *in vivo* and *in vitro*.

### circ-03955 Knockdown Inhibited the Occurrence of EMT in Osteosarcoma

Previous studies have shown that circ-03955 knockdown could inhibit the proliferation, apoptosis, invasion, and migration of osteosarcoma. In order to further investigate the effects of circ-03955 on EMT on osteosarcoma, we measured the expression of E-cadherin and N-cadherin in circ-03955 knockdown tissues and cells. Compared with sh-NC, E-cadherin was obvious in the cytoplasm of the sh-circ-03955 group. N-cadherin was also expressed at lower levels in the cytoplasm of sh-circ-03955 (**Figure 2G**). The Western blot results confirmed that, compared with sh-NC, the expression of E-cadherin was markedly increased in the sh-circ-03955 group, and the expression of N-cadherin was significantly decreased (all *P <* 0.001) (**Figure 2H**). The same results were also found in tissues. The immunohistochemical results show that the intracellular deposition of E-cadherin protein in the sh-circ-03955 group was significantly higher than that in the sh-NC group, and the intracellular deposition of N-cadherin protein was significantly reduced (**Figure 2I**). Western blotting also confirmed that, compared with sh-NC, the expression of E-cadherin was increased, and N-cadherin was decreased in sh-circ-03955 (all *P <* 0.001) (**Figure 2J**). These results indicate that circ-03955 knockdown could effectively inhibit the EMT of osteosarcoma *in vivo* and vitro.

### miR-3662 Mimics Inhibit Osteosarcoma Cell Proliferation, Promote Cell Apoptosis, and Inhibit Cell Invasion

**Figure 3:**
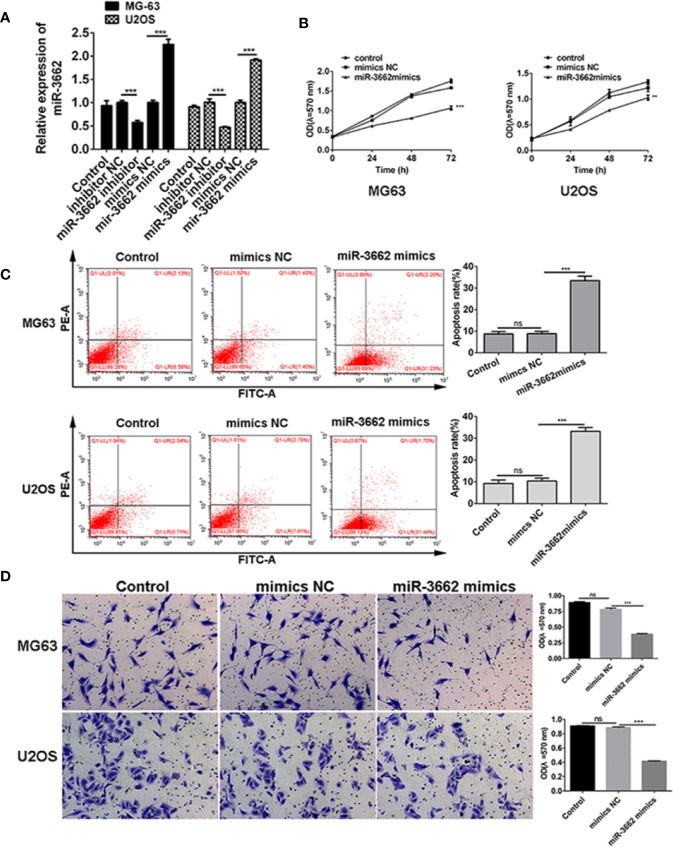
miR-3662 mimics inhibit osteosarcoma cell proliferation, promote cell apoptosis, and inhibit cell invasion. **(A)** qRT-PCR was used to verify the transformation efficiency of miR-3662 mimics and inhibitor in osteosarcoma cells MG-63 and U2OS. **(B)** MTT assay was used to detect the effects of miR-3662 mimics on the proliferation of osteosarcoma cells MG-63 and U2OS at 24, 48 and 72 hours after transfection. **(C)** Flow cytometry was used to detect the effect of miR-3662 mimics on apoptosis of osteosarcoma cells MG-63 and U2OS at 48 hours after transfection. **(D)** Transwell assay was used to detect the effects of miR-3662 mimics on the invasion of osteosarcoma cells MG-63 and U2OS at 48 hours after transfection. Data are presented as the mean ± standard deviation of three independent experiments, ns, not significant, **p < 0.01, ***p < 0.001 vs. shNC group.

Previous studies show that the expression of miRNA-3662 is low in osteosarcoma tissues and cells (**Figures 1I, J**). To verify the role of miRNA-3662 in the development of osteosarcoma, miRNA-3662 mimics and inhibitor were transfected into osteosarcoma cells MG63 and U2OS (**Figure 3A**). MTT results show that, compared with NC mimics, the proliferation of MG-63 and U2OS cells in the miR-3662 mimics group was significantly decreased (*P <* 0.001, *P <* 0.01) (**Figure 3B**). Flow cytometry showed that, compared with the mimics NC group, the apoptotic rate of MG-63 and U2OS cells in the miR-3662 mimics group was significantly increased (*P <* 0.001) (**Figure 3C**). Transwell test results showed that, compared with the mimics NC group, the invasion number of the MG-63 and U2OS cells in the miR-3662 mimics group decreased significantly (*P <* 0.001) (**Figure 3D**). These results indicate that miR-3662 mimics could effectively inhibit the proliferation and invasion and promote the apoptosis of osteosarcoma.

### circ-03955 Acts as a ceRNA by Sponging miR-3662 and Indirectly Regulates MTDH Expression

**Figure 4:**
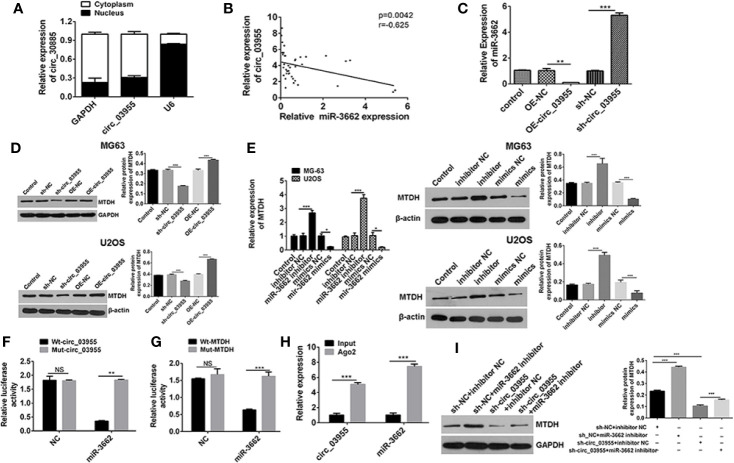
Circ-03955 acts as a ceRNA by sponging miR-3662 and indirectly regulates MTDH expression. **(A)** The differential expression of circ-03955 in MG-63 cytoplasm and nucleus of osteosarcoma cells verified by qRT-PCR. **(B)** Analysis of the correlation between circ-03955 and miR-3662 expression in osteosarcoma. **(C)** qRT-PCR was used to detect the effect of OE-circ-03955 and sh-circ-03955 on the expression of miR-3662 in osteosarcoma MG-63 cells 48 hours after transfection. **(D)** Western blotting was used to detect the effects of OE-circ-03955 and sh-circ-03955 on the expression of MTDH protein in osteosarcoma cells MG-63 and U2OS at 48 hours after transfection. **(E)** The expression of MTDH in osteosarcoma MG-63 and U2OS was detected by qRT-PCR after 48 hours of transfection. Western blotting was used to detect the expression of MTDH protein in osteosarcoma MG-63 and U2OS at 48 hours after transfection. **(F)** The binding relationship between circ-03955 and miR-3662 was detected by the dual luciferase reporting system. **(G)** The binding relationship between miR-3662 and MTDH was detected by the dual luciferase reporting system. **(H)** Ago2-RIP assays were used to detect the relationship between circ-03955 and miR-3662. **(I)** Western blot assays were used to verify that circ-03955 reverses the inhibitory effect of miR-3662 on MTDH. Data are presented as the mean ± standard deviation of three independent experiments, NS, not significant, *p < 0.05, **p < 0.01, ***p < 0.001.

Previous analyses confirm that miR-3662 is the target gene of circ-03955, and MTDH is the target gene of miR-3662. In order to verify the results of the bioinformatics analysis, we carried out follow-up studies. qRT-PCR results showed that circ-03955 was expressed primarily in the cytoplasm (**Figure 4A**). The expression of circ-03955 was negatively correlated with that of miR-3662 (*r* = −0.625, *P* = 0.0042). To verify the effect of circ-03955 on the expression of miR-3662 and MTDH, OE-circ-03955 or sh-circ-03955 were transfected into MG-63 cells. The qRT-PCR and Western blot results showed that circ-03955 overexpression can inhibit the expression of miR-3662 and promote the expression of MTDH. The knockdown of circ-03955 was shown to promote the expression of miR-3662 and inhibit the expression of MTDH (**Figures 4C, D**). miR-3662 mimics can also inhibit the expression of MTDH, whereas miR-3662 inhibitor can promote the expression of MTDH in osteosarcoma cells from the MG-63 and U2OS cell lines (**Figure 4E**). To further investigate the relationships between circ-03955 and miR-3662 and miR-3662 and MTDH a dual luciferase reporting system was used, and the results showed that circ-03955 was directly targeted by miR-3662 (**Figure 4F**), and MTDH was directly targeted by miR-3662 (**Figure 4G**) in MG-63 cells.

Ago2, as an effector protein of RISC, participates in miRNA assembly and degradation. Therefore, when the Ago2 protein was pulled down, the circRNA and miRNAs bound to RISC were also pulled down in the RIP assay (PMID: 30999843). The RIP test was performed with Ago2 as the antibody to research the relationship between circ-03955 and miR-3662. We found that circ-03955 and miR-3662 were preferentially enriched in the Ago2 pellet relative to control IgG immunoprecipitates, and endogenous circ-03955 pull-down was specifically enriched in miR-3662-transfected cells (**Figure 4H**). Knockdown of circ-03955 inhibited the increase of MTDH expression induced by the miR-3662 inhibitor (**Figure 4I**). These results suggest that circ-03955 functions as a ceRNA by sponging miR-3662 and indirectly regulating MTDH expression.

### miR-3662 Can Reverse the Effects of circ-03955 or MTDH on Osteosarcoma Apoptosis and EMT

**Figure 5:**
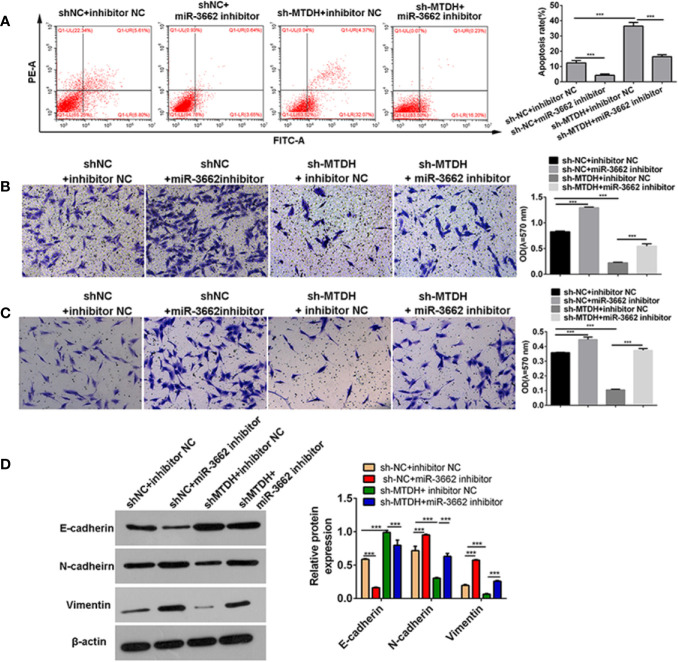
miR-3662 inhibitor rescues the inhibitory effect of shMTDH on osteosarcoma cells. **(A)** Flow cytometry was used to detect the apoptotic rate of the four groups in the recovery experiment and to verify that miR-3662 could reverse the apoptotic effect of MTDH on osteosarcoma cells. **(B)** Transwell assays were used to detect the migration of four groups in the recovery experiment and to verify that miR-3662 could reverse the migration effect of MTDH on osteosarcoma cells. **(C)** Transwell assay was used to detect the invasion of the four groups in the recovery experiment and to verify that miR-3662 could reverse the invasion effect of MTDH on osteosarcoma cells. **(D)** Western blotting was used to detect the expression of EMT-related proteins (E-cadherin, N-cadherin, and Vimentin) in four groups and to verify that miR-3662 could reverse the effect of MTDH on the EMT of osteosarcoma cells. Data are presented as the mean ± standard deviation of three independent experiments, ***p < 0.001.

**Figure 6:**
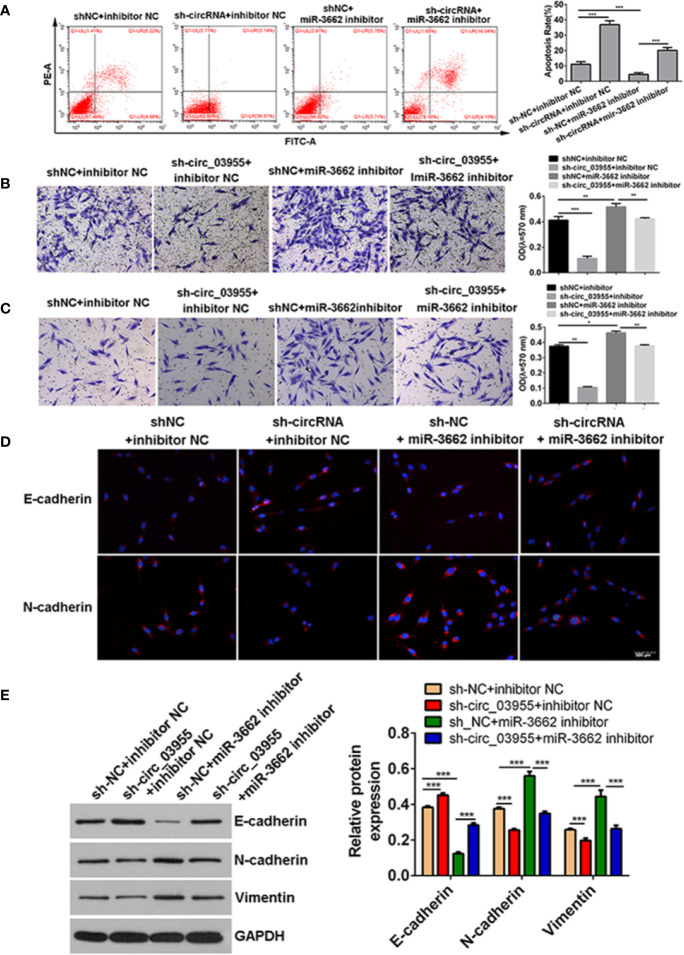
shcirc_03955 rescues the inhibitory effect of miR-3662 on osteosarcoma cells. **(A)** Flow cytometry was used to detect the apoptotic rate of the four groups in the recovery experiment and to verify that miR-3662 could reverse the apoptotic effect of circ-03955 on osteosarcoma cells. **(B)** Transwell assay was used to detect the migration of four groups in the recovery experiment and to verify that miR-3662 could reverse the migration effect of circ-03955 on osteosarcoma cells. **(C)** Transwell assay was used to detect the invasion of the four groups in the recovery experiment and to verify that miR-3662 could reverse the invasion effect of circ-03955 on osteosarcoma cells. **(D)** The expression of E-cadherin and N-cadherin proteins in four groups was detected by immunofluorescence assay. (Scale bar = 500 μm). **(E)** Western blotting was used to detect the expression of EMT-related proteins (E-cadherin, N-cadherin, and Vimentin) in four groups and to verify that miR-3662 could reverse the effect of circ-03955 on the EMT of osteosarcoma cells. ***P < 0.001 indicated that the difference has statistical significance. Data are presented as the mean ± standard deviation of three independent experiments, *p < 0.05, **p < 0.01, ***p < 0.001. The red color indicates the expression of the target protein, and the blue color indicates the nuclear staining. The magnification is 200×.

Previous studies show that circ-03955 can inhibit the progression of osteosarcoma and the occurrence of EMT and regulate MTDH expression *via* miRNA-3662. It is reported that MTDH can promote the occurrence of EMT in osteosarcoma (21). To explore whether circ-03955 exerts its functions by miR-3662/MTDH in osteosarcoma, rescue experiments were performed. Downregulation of MTDH significantly enhanced cell apoptosis and inhibited cell migration and invasion, and the effect could be abolished by the miR-3662 inhibitor (**Figures 5A–C**). Downregulation of MTDH also significantly inhibited Vimentin and N-cadherin expression and promoted the expression of E-cadherin, an effect that could be inhibited by the miR-3662 inhibitor (**Figure 5D**). Downregulation of circ-03955 significantly promoted the apoptosis of MG63 cells and inhibited cell migration and invasion, also effects that could be inhibited by the miR-3662 inhibitor (**Figures 6A–C**). Downregulation of circ-03955 markedly inhibited the expression of Vimentin and N-cadherin and promoted the expression of E-cadherin, and the effect could be inhibited by miR-3662 inhibitor (**Figures 6D, E**). These results indicate that circ-03955 contributes to EMT by suppressing miR-3662.

## Discussion

Osteosarcoma ranks third in the incidence of malignant tumors in children and adolescents and in mortality rate ranks first (22, 23). In the past few decades, despite improvements in surgical and adjuvant radiotherapy techniques, the five-year survival rate of osteosarcoma patients with metastasis or recurrence has not improved, remaining at about 20% (24, 25). Due to the low five-year survival rate of osteosarcoma, it is important to explore new treatment programs for clinical application. Recently, circRNA has been shown to play an important role in the progression of many diseases and appears to be abnormally expressed in various types of tumor, providing a potential novel therapeutic target for tumor treatment (26–28). In this study, we identified a novel circRNA (circ-03955) and confirmed that circ-03955 was upregulated in osteosarcoma tissues and cells. Circ-03955 knockdown inhibited the proliferation, migration, and invasion of osteosarcoma cells from the MG63 and U2OScell lines and promoted apoptosis. Knockdown of circ-03955 inhibited the progression of osteosarcoma in a mouse model. These results suggest that circ-03955 acts as a tumor promotor in osteosarcoma.

With the increase in circRNA research, some circRNA-related bioinformatics databases have emerged. The CircNet database reports that 212,950 circRNAs have been found. In the nc2Cancer database, 172 circRNA have been reported to be related to 31 malignant tumors. Based on these databases, some novel circRNAs have been screened out by bioinformatic methods, and their function in tumors has been verified. Liu (29) used the CircNet database to establish that the expression of circ-ZEBl.5, circ-ZEBl.19, circ-ZEB1.17, and circ-ZEB l.33 in lung cancer was lower than that in normal tissues. It has been demonstrated that circ-EIF3J, circ-PAIP2, and circ-FUNDCl are significantly overexpressed in cervical cancer cells (30). In this study, we identified a differentially highly expressed circ-03955 form GSE99671 data set using bioinformatics methods. Circ-03955 is a novel circular RNA, located in chr8 of hg19 with a genomic length of 286 bp. However, no studies have suggested that circ-03955 contributes to any disease, including osteosarcoma. We found that circ-03955 was circular RNA using qRT-PCR after RNase R treatment. We propose for the first time that circ-03955 is a promotor of osteosarcoma. We also found that circ-03955 has a marked effect on the EMT of osteosarcoma. Thus, circ-03955 was established as a circRNA with the potential to be a target for further experiments. miR-3662, which is a target of circ-03955, was shown to be significantly decreased in osteosarcoma. A previous study showed that miR-3662 is downregulated in acute myeloid leukemia, whereas it is increased in lung adenocarcinoma (31). However, the role of miR-3662 and related mechanisms were unclear in osteosarcoma. Our study showed that miR-3662 was downregulated in osteosarcoma, and cell function was contrary to circ-03955. We explored the interactions between circ-03955, miR-3662, and MTDH, which is the target of miR-3662.

In 2013, Hansen (32) and Memczak (33) found that circRNA can act as a “sponge” to block and competitively inhibit miRNAs in tumors, so circRNA can participate in the pathogenesis of tumors as an endogenous competitive RNA. There are more than 60 conservative binding sites on the sequence of circular RNA-7 (ciR-7) binding to miRNA-7, and they are abundant in the cytoplasm. They can bind to more than 20,000 miRNAs-7 in cells, thus affecting the transcriptional inhibition function of miRNA-7 in cells (33, 34). CircRNA SRY has been found to have six binding sites, thus acting as a sponge for miRNA-138 (35). Studies show that circRNA can competently inhibit miRNAs to participate in the signaling pathway of tumors (36, 37). There are many studies that have proposed a sponge mechanism between circRNA and miRNA. Our results indicate that there is a sponge mechanism between circ-03955 and miR-3662. We found that the expression of circ-03955 and miR-3662 was negatively correlated in clinical samples of osteosarcoma tissues and cells. Circ-03955 could bind to miR-3662, and miR-3662 could bind to MTDH. circ-03955 and miR-3662 regulate the effect of MTDH on osteosarcoma through endogenous competition. Our study confirmed that circ-03955 acts as a sponge to competitively inhibit miR-3662 in osteosarcoma, and downstream target MTDH was regulated by miR-3662. This is consistent with the idea that circRNA can participate in the signaling pathway of tumors through competitive inhibition of miRNAs.

Recent studies have shown that MTDH could promote lung metastasis of breast cancer (38). Other studies have revealed that MTDH plays an important regulatory role in tumor proliferation, differentiation, apoptosis, migration, invasion, and drug resistance (39–41) *via* the Ha-ras, PI3K/Akt, NF-kappa B, and Wnt/beta-catenin signaling pathways (38, 41, 42). MTDH is the target gene of miR-630. Zhao pointed out that miR-630 can directly target the MTDH gene. In the MDA-MB-231-luc and BT549 breast cancer cell lines, high expression of miR-630 can inhibit cell movement and invasion (43). However, whether other non-coding RNAs, such as circRNA, play a regulatory role in MTDH expression has not been ascertained. Our study screened out the miR-3662 that participates in MTDH regulation and can upregulate circRNA, and the upregulated circRNA was circ-03955. Subsequently, we validated the inhibitory effects of circ-03955 on MTDH by reversing the inhibitory effect of miR-3662 through recovery experiments. miR-3662 can reverse the promotion of MTDH on osteosarcoma, and circ-03955 can reverse the inhibition of miR-3662 on osteosarcoma, according to the recovery experiments. It was further demonstrated that circ-03955 and miR-3662 compete to regulate the expression of MTDH, thereby affecting the proliferation, apoptosis, and EMT of osteosarcoma.

In conclusion, our study shows that circ-03955 is highly expressed in osteosarcoma. Circ-03955 knockdown can inhibit the proliferation of osteosarcoma cells, promote cell apoptosis, and inhibit cell migration and invasion. Further analysis showed that circ-03955 was negatively correlated with miR-3662 in osteosarcoma, and circ-03955 could bind to miR-3662, indicating that there was a competitive relationship between circ-03955 and miR-3662 in the cell. Circ-03955 competes with miR-3662 to regulate the expression of MTDH and then affect the progression and occurrence of EMT in osteosarcoma. These findings provide a basis for potential therapeutic targets for the treatment of osteosarcoma.

## Data Availability Statement

The datasets presented in this study can be found in online repositories. The names of the repository/repositories and accession number(s) can be found in the article/supplementary material.

## Ethics Statement

The animal study was approved by the medical ethics committee of the third Xiangya Hospital, central South University.

## Author Contributions

YZ designed the work and offered funds. ZW and MD designed the work, performed the experiment, and submitted the manuscript. LC and WW performed the experiment and collected the data. GL, DL, and ZH analyzed data and modified the manuscript. All authors contributed to the article and approved the submitted version.

## Funding

This study was supported by the New Xiangya Talent Project of the Third Xiangya Hospital of Central South University (Grant NO. JY201516).

## Conflict of Interest

The authors declare that the research was conducted in the absence of any commercial or financial relationships that could be construed as a potential conflict of interest.
